# The complexity and cost of vaccine manufacturing – An overview

**DOI:** 10.1016/j.vaccine.2017.06.003

**Published:** 2017-07-24

**Authors:** Stanley Plotkin, James M. Robinson, Gerard Cunningham, Robyn Iqbal, Shannon Larsen

**Affiliations:** a University of Pennsylvania and Vaxconsult, USA; b Independent consultant, USA; c Founder and Principal Consultant with Innovations for Global Health (iGH), LLC, USA; d Bill & Melinda Gates Foundation, PO Box 23350, Seattle, WA 98102, USA

**Keywords:** Vaccines, Manufacturing, Manufacturing costs, Gavi, Quality, Licensure, WHO, Prequalification, UNICEF

## Abstract

As companies, countries, and governments consider investments in vaccine production for routine immunization and outbreak response, understanding the complexity and cost drivers associated with vaccine production will help to inform business decisions. Leading multinational corporations have good understanding of the complex manufacturing processes, high technological and R&D barriers to entry, and the costs associated with vaccine production. However, decision makers in developing countries, donors and investors may not be aware of the factors that continue to limit the number of new manufacturers and have caused attrition and consolidation among existing manufacturers. This paper describes the processes and cost drivers in acquiring and maintaining licensure of childhood vaccines. In addition, when export is the goal, we describe the requirements to supply those vaccines at affordable prices to low-resource markets, including the process of World Health Organization (WHO) prequalification and supporting policy recommendation. By providing a generalized and consolidated view of these requirements we seek to build awareness in the global community of the benefits and costs associated with vaccine manufacturing and the challenges associated with maintaining consistent supply. We show that while vaccine manufacture may prima facie seem an economic growth opportunity, the complexity and high fixed costs of vaccine manufacturing limit potential profit. Further, for most lower and middle income countries a large majority of the equipment, personnel and consumables will need to be imported for years, further limiting benefits to the local economy.

## 1 Introduction

Despite the market dominance of vaccine manufacturers based in high and middle-income countries, there are many reasons why a low-income country or regional grouping of countries may want to establish their own vaccine supply [1]. These include: supply security, control over production scheduling and sustainability, control of costs, socio-economic development, and rapid response to local epidemics including emerging infectious diseases. Where Expanded Program on Immunization (EPI) vaccines are provided, vaccine uptake has increased and childhood morbidity and mortality have fallen [2]. Given the importance of vaccines in public health programs, governments and donors have invested in vaccine R&D and production in low-resource settings [3]. However, there are many factors to consider prior to commitment to this capability – the high failure rate of preceding efforts [1]; the high cost and time required to establish complex processes, and capabilities for production of a broad portfolio of vaccines [4]; fragmented or inconsistent demand [5]; diverse regulatory requirements; and limited local competence and experience [6]. Additionally, to produce at low cost requires strategic commercial planning and adoption of various cost saving approaches. While some manufacturers have successfully produced vaccines for decades, others have faltered or failed, and relatively little information is available in the literature on the challenges, complexity and cost of vaccine manufacturing. This paper consolidates information from disparate sources to begin to fill this void and to drive better understanding of the costs associated with robust vaccine production capabilities.

## 2 Vaccine manufacturing overview

Vaccine manufacture is one of the most challenging industries. Even the most basic manufacturing steps necessary to produce vaccines in a manner that is safe, effective, and consistent over the life cycle of a vaccine are difficult to execute [7]. Outcomes can vary widely due to the nearly infinite combinations of biological variability in basic starting materials, the microorganism itself, the environmental condition of the microbial culture, the knowledge and experience of the manufacturing technician, and the steps involved in the purification processes. To add to the complexity, the methods used to analyze the biological processes and antigens resulting from vaccine production often have high inherent variability. Failure to manage these risks can result in costly product recalls, and suspensions and penalties may be assessed if a manufacturer fails to fulfil supply agreements. In addition, lack of supply can disrupt routine immunization programs and negatively impact national public health outcomes.

Regulatory authorities license not only a specific biological entity, but also the processes by which that entity is produced, tested, and released for use. Subtle changes in the production process may alter the final product and change its purity, safety, or efficacy. Further, the *in vitro* analytics required to release the product may not detect a change in process and a clinical trial may be needed to validate a new process and to maintain licensure of a product. This compounded risk of biological and physical variability makes vaccine manufacturing more challenging than typical small molecule pharmaceuticals and is a primary root cause of the high proportion of vaccine manufacturing failures and supply shortages [7], [8]. This is also the main reason why the number of vaccine manufacturers that succeed and thrive remains low despite unmet demand for many vaccines globally. Moreover, individual vaccine prices do not always decline, even after the patents expire, in contrast to pharmaceutical products. In fact, many vaccine patents protect the manufacturing process rather than the antigen that is produced by the process, which is not always the analogous case for small molecule pharmaceutical products. These process patents may present a more significant barrier to entry than the patent on the vaccine composition itself.

### 2.1 Process development and maintenance

Significant changes in the manufacturing process, such as new facilities, manufacturing equipment or changes in raw materials, will typically trigger new regulatory requirements, including clinical trials. These requirements will confirm that the vaccine is still effective and comparable to the product produced by the original vaccine process and studied in the original clinical studies. As this is a significant obstacle for continuous process improvement and process modernization for vaccine manufacturers, it is optimal to have visibility into how the product will be made at commercial scale early in the development process. This prevents having to maintain a suboptimal manufacturing process for the long life-cycle of the vaccine. Emphasis on process development is a major success factor in being first to market with new biopharmaceuticals and inadequate process development is often implicated in late stage product development failures [9], [10]. Manufacturers are challenged to balance the competing goals of speed to market and process optimization; getting to market earlier increases revenue in the short term, but locking in a further optimized process may generate cost savings over the entire vaccine life-cycle.

### 2.2 Life cylce and lead time

Most vaccines have a long life-cycle; some vaccines used today were developed in the 1940s and 1950s and remain essentially unchanged [7]. To maximize a vaccine’s life-cycle, raw material and component supplies must be available and consistent in composition for decades. Optimal and efficient process development requires a sustained supply of quality raw materials from reliable vendors. Competitive pressure from other industries for the same materials can increase cost and interrupt supply. Likewise, production processes may need to be adapted as technologies advance and production components (e.g., filters and resins) change over time.

The lead time to produce a vaccine lot ranges from several months (e.g., influenza vaccine) to three years [8] (e.g., pentavalent and hexavalent combination vaccines) and vaccine shelf-life generally ranges from one to three years. The vaccine must conform with release specifications for the duration of manufacturing and storage, and stability of the product must be confirmed through long-term stability studies. Even when vaccines have been licensed, several lots are tested for stability each year to confirm that any process changes made did not have a deleterious effect. Stabilization may be achieved simply by managing pH with the appropriate buffer preparations, or for products that are inherently unstable such as some live viral vaccines, by lyophilizing (freeze-drying) to remove water. Lyophilizing creates a dry form that is less likely to degrade and can be reconstituted shortly before use. In extreme cases, formulation requirements alone can increase costs several-fold and in the case of lyophilization, may reduce capacity considerably while adding significant capital and operating cost.

### 2.3 Production facilities and equipment

**Fig. 1:**
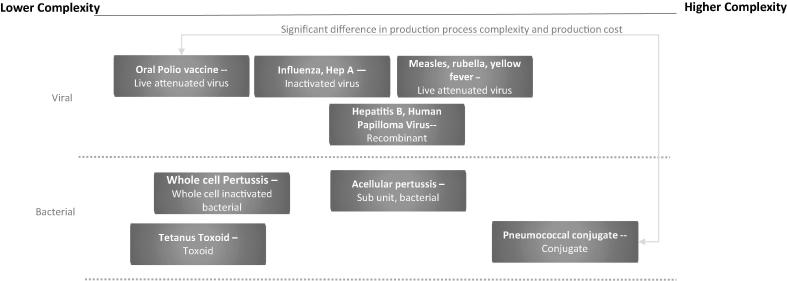
Examples of vaccines and relative production complexity.

There are many production platforms in use today and they vary widely [7], [11]. Fig. 1 shows the range and relative production complexity of various vaccines and vaccine types [12]. At one end is live attenuated oral polio vaccine with significantly lower Cost of Goods Sold (COGS) while at the other end is the highly complex pneumococcal conjugate vaccine [13]. While there may be common equipment across platforms such as bio reactors, filtration and chromatography equipment, filling and lyophilization equipment, the sequence of operations and the specific cycles for each product vary. In most cases, each product (or group of products within a product family) has its own dedicated facility and production team. This dedicated labor and equipment allows for flexibility to address unpredictable demand, but tends to increase costs.

### 2.4 Product portfolio

Most manufacturers aspire to license multiple products, creating a vaccine portfolio which mitigates the high fixed costs of manufacturing and volatility in global markets, but also presents formidable production challenges. Including the research phase, estimates of time required to bring a product to the international market range from 5 to 18 years [14]. Managing all products simultaneously without incident requires skill, experience and knowledge that has to-date only been mastered by a select group of companies and national suppliers. Nevertheless, production shortfalls still occur even with the most capable manufacturers. Successful manufacturers typically develop competencies and pursue production of additional and more complex vaccines over time.

## 3 Production economics

### 3.1 Cost categories and accounting

There are many costs that influence investment, development and pricing of vaccines and accounting can be complex. This article focuses on manufacturing costs and related items as a sub-set of the overall expense of vaccine production. A brief discussion of costs of clinical testing is included, but is not the focus of this article. As accounting practices vary widely we use a standardized methodology [15] to describe cost of goods, allowing for comparison across multiple sources. The major cost categories discussed below are facilities and maintenance, raw materials and production, personnel and quality assurance/compliance. The cost of raw materials, components, labor, analytics, and documentation of the process and assay results comprise the direct costs of vaccine manufacturing. Indirect costs include the creation and management of the quality systems, production planning, QC planning, warehousing and distribution, inventory management and overhead functions such as regulatory, sales, marketing and management.

Most National Regulatory Authorities (NRAs) require documentation of Current Good Manufacturing Processes (cGMP) – which can increase the production manpower requirement [16] as the number of Quality Assurance personnel may be one-half of the number of production workers, depending on the analytics and number of products and raw materials [12]. Typically, vaccine manufacturers use proven technologies, equipment, and processes to maintain a low-risk approach to development and manufacturing.

Various valid accounting methodologies exist and are used for different reasons. The appropriate inclusion and allocation of costs when multiple markets are served is a topic of intense debate. There are often differences of opinion on whether fixed or sunk costs, such as research and development or facilities, should be included. Considering only marginal or variable costs of production will lead to a lower calculated COGS, and does not accurately represent the full cost incurred by a manufacturer to bring that vaccine to market. Using a fully loaded cost basis can provide a more accurate estimate of total costs for the manufacturer for that market segment. To understand costs for each vaccine, relevant portions of each cost can be uniquely allocated to a specific vaccine and market (e.g., based on market segments are served vaccines or presentations used, or funding sources for each specific market/product). Table 1 identifies cost ranges and opportunities to reduce COGS for each cost category.

**Table 1:** Major cost drivers, impact on COGS and options to options to reduce COGs.

Cost Driver	Major Cost Driver	Relative Impact of Cost Driver on overall costs	Cost range	Options to Reduce COGS	Potential Impact of COGS Reduction Strategy	Examples
Product Development	– R & D laboratories – R & D personnel	– High fixed costs/possible to be shared across antigens	>500 M USD Risk adjusted* cost of 135 to 350M USD *Risk adjusted cost incorporates the cost and probabilities of moving to the next phase of development [14]	– Copy originator process post patent expiration	– High	– MR vaccine copied from originator vaccine
				– Perform tech transfer with established product	– High	
				– Leverage correlates of protection to avoid large efficacy studies	– Medium	
				– Purchase antigens and execute form/fill as a means of gaining experience prior to full manufacturing end-to-end	– Medium	– OPV bulk can be sourced from an approved manufacture and formulated/filled

Facilities and Equipment	Capitalized costs that depreciate over time – Land – Buildings – Machinery Ongoing costs of upkeep – Repairs – Maintenance – Utilities	– High fixed costs/design for minimizing maintenance and utilities	50 to 700M USD Example: It took Pfizer five years and 600M USD to build a manufacturing site in the US [17]	– Design for very high facility utilization. Limit the number of production platforms; force fit new processes into established platforms to reduce need for new facilities; increase utilization of existing facilities. Use multi-dose vials.	– High	– Share filling lines across multiple vaccines, when possible
						– Shift production volumes to multi-dose vials to reduce filling costs (at the risk of higher vaccine wastage in field).
				– Use single-use disposable systems to reduce capital cost	– Medium	– Reduced capital offset by higher operating (consumable) costs
				– Minimize classified production space with closed systems and RABs	– Low/Med	
				– Limit automation and process/equipment		
				– Leverage blow-fill-seal (BFS) filling technology to shrink clean room footprint and reduce final product component costs, and reduce labor		
				– Utilize Contract Manufacturing Organizations (CMO) for low volume products or until demand supports facility construction.	– Low/Med	– Seasonal influenza vaccines produced at a CMO. Reduced capital offset by CMO contract fees.

Direct Labor	Employee costs directly attributable to a specific vaccine – Wages – Benefits	– Low/typically less than 25% of total manufacturing costs	Costs can be significantly lower in China and India (25% lower for some manufacturers) of but manpower efficiency may be 120-130% of Western standards The difference is shrinking due to increasing labor costs as the requirements of cGMP practices increase	– Increase automation and single-use production technologies (balancing with potential increase in equipment or consumables costs)	– Medium	– Single-use, or disposable, bioreactors reduce cleaning and sterilization requirements, and complexity of qualification and validation
						– Pneumococcal conjugate vaccine assays are streamlined across multiple serotypes.
				– Standardize and streamline processes across as many steps and vaccines as possible.	– High	
				– Develop capacity progressively through reverse integration (packaging purchased products, filling and packing purchased products, form/fill/pack purchased products, then production of bulk drug substance for internal form/fill/pack)		
		

Overhead	Management, quality systems, IT systems	– High if company has few products – Low if overhead can be allocated across multiple products	Up to 45% of the cost of raw materials and labor combined [18]	– Invest in quality systems that can streamline quality practices and reduce costs over the long term	– Medium	– Introduce enterprise quality management software (EQMS)
				– Ensure management team has broad expertise to be leveraged across a portfolio of vaccines		

Licensing/Regulatory and commercialization	Expenses paid for the right to use product-related IP (technology) Expenses to comply with regulatory requirements to produce either for domestic market or export	– Low if experienced teams are engaged early to prepare facilities and processes for regulatory review – High if review process requires considerable rework or if delays result in lost revenue	In addition to staff and consulting costs, the new WHO process assesses the following fees: A site audit fee of 30K USD and for: Simple/Traditional vaccines: – Evaluation fee of 25 to 100K, and Annual fees of 4.8K to 140K USD Combo or Novel Vaccines: – Evaluation fee of 66.5 to 232.8K USD, and Annual fees of 8.4 to 250K USD [19].	– Pursue WHO PQ as required by UNICEF/PAHO only when intending to access markets for which they procure (e.g., Gavi)		
				– Request royalty reductions or waivers for vaccine sold in low income countries (LICs)	– Low	– Royalty for HPV antigens waived for volumes sold in Gavi
				– Produce reagents in-house or seek viable alternatives rather than license.	– Medium	– CRM produced in-house to avoid licensing cost.
				– Differentiate originator production processes sufficiently to be considered a novel process		
				– Accelerate approval by seeking NRA or WHO priority review for vaccines for neglected diseases or emergency use.	– High	
				– Utilize FDA priority review vouchers for another product and allocate savings to the vaccine that secured the voucher.	– High	– Apply priority review voucher to a product intended for high income markets to maximize the value of accelerated approval

Two commercial models of vaccine supply represent contrasting approaches to the challenge of right-sizing facility design and reducing the burden of fixed costs. Local/national vaccine producers may have good insights into population trends, including size of birth cohorts, and can readily predict domestic market size. If the population served by a local producer is small, the costs associated with vaccine development and facility construction are spread over a smaller number of doses, leading to high per-unit production costs. Larger manufacturers often plan facilities based on a commercial plan to supply multiple markets (e.g., additional countries or broader income-based market segments). Large volumes support a lower cost of goods, diluting high fixed costs over many doses. However, failure to correctly predict competition and demand across multiple markets can lead to overcapacity and may increase costs due to lower-than-optimal utilization of manufacturing facilities and supporting cost centers.

When planning production to supply multiple markets, manufacturers must consider the competitive landscape and what market share is realistic and sustainable. From a procurement perspective, the healthiest markets that best balance price affordability and sustainable production include multiple manufacturers whose collective capacity exceeds total demand, but not by so much that each manufacturer is severely underutilizing their facilities. A monopoly supplier may achieve the lowest possible costs through maximum volumes, but international agencies and donors may consider supply security at risk and actively encourage new suppliers to enter the market. In contrast, in a market supplied by many manufacturers with good supply security but high excess capacity, it is likely that some manufacturers will produce at low volumes relative to their individual capacity and experience higher than anticipated costs.

### 3.2 Product development

The clinical requirements for vaccine development are generally well understood, although they vary by disease target. Requirements can be particularly challenging for an innovative vaccine type, or new disease area, since early on the developer may have little knowledge of the exact mechanism of action for the vaccine or the vaccine’s impact on disease. This paper focuses on the steps of development and validation of the manufacturing process leading to a licensed product. The key steps for process and analytical development and associated time frames during clinical development are outlined in Table 2.

**Table 2:** Key vaccine development stages and process/system expectations.

Stage	Objective	Process development and manufacturing
Exploratory & Pre-clinical	Assess safety and immunogenicity of a target antigen or cell in cell culture or animal disease models; assess a safe starting dose for human clinical studies	Small scale, often crude extracts or purchased antigens. The cost of manufacturing generally is not critical at this stage, although method of manufacturing is critical to the character of the ultimate product. Process development is often delayed until after some proof of concept in animal models is confirmed
Clinical Trial Authorization Application	Apply for approval to conduct human clinical studies	Outline all critical manufacturing steps and analytical methods used to produce and release the product and placebo, including all reagents, components, specifications, acceptable limits to manufacture and release the product ensuring the identify, strength, quality, and purity. Demonstrate stability of the drug product and placebo for at least the duration of the clinical studies
Phase I Vaccine Trials	Assess the safety of the candidate vaccine; determine the type and extent of immune response that the vaccine provokes	All human clinical materials are recommended to be made under cGMP. The state of the process development varies with strategy; complete process optimization is often deferred until after proof of concept in humans, but all process changes need to be qualified prior to advancing to the next clinical stage and deferring development can delay the next stages or risk the vaccine failure for unforeseen or unintended consequences of these changes
Phase 2 Vaccine Trials	Assess candidate vaccine safety, immunogenicity, dose response, schedule of immunizations, and method of delivery	Prior to initiating phase 2 studies it is recommended that all major process changes are incorporated and qualified. Significant changes after this step can risk repeating phase 1/2 studies. Projected cost of goods is confirmed to be appropriate for the intended use and markets
Phase 3 Vaccine Trials	Assess the candidate vaccine in the target populations for safety and rare adverse events Vaccine efficacy is estimated. Vaccine manufacturing consistency is confirmed. Concomitant testing with other prescribed vaccines may be required	Processes are finalized and validated. Analytical tests for manufacturing and release are completed and validated. Costs of goods are confirmed to be appropriate for the intended use (as changes to reduce costs would need to be re-validated and may require additional clinical testing)
Approval & Licensure	Submit and gain approval of the Biological Product Application	Full review and documentation of the manufacturing methods and analytical methods for licensed production; full shelf-life stability studies completed and in specification; completed process validation, facility validation, release testing validation; development of production and release protocols; launch lots prepared and released. Agency inspection of all manufacturing and release facilities and documentation of all manufacturing and quality systems
Post-Licensure Monitoring	Confirm filed use of vaccine is consistent with expectations from the clinical studies and finalized manufacturing and release process	Routine (annual, biennial) agency inspections of manufacturing and testing facilities. Annual product review and reporting demonstrating the process remains in control
License Amendments	Confirm any changes to the intended use of the vaccine in different populations or and changes to the manufacturing process (seeds, raw material sources, process steps, release steps, equipment, facilities, etc.) do not adversely affect the product purity, safety, or effectiveness	Process improvements after license approval are expected to keep the process optimized and to take advantage of advances in science and manufacturing methods, but can be expensive and risky (unintended consequence of a change). Significant changes should be carefully considered with respect the risk/benefit of the change

There is wide variability in the costs of process and analytical development, manufacturing, and documentation is dependent on vaccine type, innovation level, disease target and regulatory body. A broad survey of industry indicates that the Chemistry, Manufacturing and Controls (CMC) development for a vaccine exceeds 50 M USD and consumes more than 80 person-years in human resources [20]. Phase 1 development can range from 2 M USD for a translational product to 60 M USD for an innovative product, with an average cost of Phase 1 of 12 M USD for CMC elements [21]. Despite the difficulty of quantifying costs, one risk-adjusted estimate of R&D costs are 135–350 M USD [14]. Another report suggests that the total costs of vaccine development can range from 200 to 500 M USD [22] and can take 15 years or more [21]. Manufacturers from developing countries may choose to obtain technology from major multinational manufacturers, which obviates the cost and development time inherent in research and development. This strategy enables lower prices for vaccines, which is appealing to public health authorities in those countries.

### 3.3 Facilities and equipment

The vaccine manufacturing facility represents a significant fixed and ongoing maintenance cost for the vaccine manufacturer. Traditionally manufacturers focus on identifying installed capacity for a particular production process to serve a specific market need. This requires careful assessment of market opportunity to determine optimal capacity and utilization. If installed capacity is too large, the additional fixed cost burden increases per-unit dose cost. Conversely, capacity that is lower than market need can lead to opportunity costs and lack of flexibility of supply as market conditions change. Even with the best planning, new technologies that change how vaccines are packaged and delivered may disrupt the industry by further segmenting the market with different product presentations of the same vaccine. For example, changing the primary containers or vials can significantly affect the production and facilities requirements. On top of costs to develop the new technology, when the product lifecycle of the original vaccine presentation is cut short or altered, full costs of the original presentation are not recovered and the manufacturer is forced to invest further to maintain market share.

Facilities can cost 50–500 M USD per antigen based on the high complexity of design, automation, segregation, utilities, and contamination controls, and as much as 700 M USD for multiple vaccines [14], [21]. cGMP space may cost 600 USD/ft^2^; non-cGMP space may cost 350 USD/ft^2^ [16]; clean rooms and containment rooms may cost more. The US Department of Defense estimated the 25-year life-cycle cost of a 3-product facility to be 1.56 billion USD and that 7 years are needed to design, build, validate, and commence commercial manufacturing [6]. This estimate is for high-resource countries, and the actual facilities cost may be much lower in low-resource countries. However, as discussed below, many other costs can be expected to be as high or higher in low-resource countries, as many materials may be imported and some key personnel may be hired from other countries as expatriates.

### 3.4 Labor (direct and overhead)

The ability to hire, train, and develop production and quality personnel to maintain the process and quality systems is a challenge even for highly experienced manufacturers. Technical competence is essential as is knowledge of the latest technologies and global regulatory requirements [16]. Globally, there is a scarcity of personnel with the requisite skills and expertise needed by the vaccine industry [6]. Vaccine production requires a deep scientific knowledge and persistent curiosity to understand and detect the subtle signals a biological process may send that are not detectable in release data. Experienced workers use caution when considering changes in processes or facilities, or when responding to process or equipment failure. Sustaining vaccine manufacturing requires developing a strong base of scientific, technical, product-specific manufacturing and quality control system knowledge. Countries such as India, Brazil and China, with large populations and sound technical and scientific education systems, have succeeded in creating a new and growing cohort of technicians and skilled workers suited for the highly-detailed work of vaccine manufacturing. New market entrants in other geographies may underestimate the difficulty of developing this type of knowledge base in tandem with a comprehensive training system.

Labor costs vary significantly by country, depending on the capabilities and education of the local workforce, the typical personnel roster for an average facility in low-resource countries will often include local and expatriate employees to secure the relevant technical skills required for vaccine production and release. Most expatriate staff will require higher total compensation and benefits than local employees, increasing the overall cost of labor and decreasing local employment opportunities.

### 3.5 Consumables

Vaccines are often produced using raw materials produced by biological production processes (e.g., yeast extract, natural or recombinant enzymes). These materials add inherent biological variability to the manufacturing or analytical processes. Due to their specialized nature, these raw materials may be limited in supply, and subject to shortages or process changes as suppliers change methods to increase productivity or their bottom line. Also, when products are derived from materials of animal origin, they carry the risk of adventitious agents which potentially can contaminate the production process. Raw materials of animal origin are subject to extensive testing for viral or other microbial contamination, and are generally sourced from regions free of certain diseases.

Materials that are in short supply are often expensive because of normal dynamics of supply and demand. Perhaps more important than cost, short supply of raw materials results in production interruptions and vaccine shortages. If a company reduces supply risk by contracting multiple suppliers for critical materials, the volumes ordered from each supplier will be reduced, likely resulting in higher prices. However, when produced locally, consumables can be an area of costs savings for vaccine manufacturers in low-resource countries, with prices estimated to be as low as 15% of those in high-resource countries [12].

## 4 Licensing/regulatory and commercialization

Regulatory and other licensing requirements are well documented. Although they are largely similar across each NRA, rules and requirements continually evolve and focus and enforcement varies. Significant events in the industry may catalyze changes in regulations or enforcement; certain lots of vaccine product may be made only for specific countries based on those regulations, increasing the complexity of logistics and limiting flexibility of inventory. NRAs may have varying license or compliance requirements, and these may be somewhat open to interpretation by the companies and the individual reviewers and inspectors [8].

A manufacturer must comply with all requirements of its NRA (and those in countries in which it wishes to market its vaccine) and adjust to changes in regulations. These requirements include routine monitoring of adverse event data, and annual reporting of specific manufacturing information (e.g., data trends, change management, stability review, critical investigations of any process failures or unexpected trends). The manufacturing facilities are subject to routine and unannounced regulatory inspections to review conformance with cGMP, maintenance of facilities, manufacturing and quality systems, and performance of the process. To export product, a specific license must be granted by the importing country, often requiring country-specific clinical trials; it is also then subject to similar compliance requirements, including routine inspections from those NRAs and global adverse event monitoring and reporting. A firm that exports product globally may need to manage scores of unique licenses for each market where the product is licensed, and is subject to nearly continuous inspection by multiple NRAs.

Beyond the licensing process, companies wishing to sell product into channels such as the United Nations Children’s Emergency Fund (UNICEF) Supply Division, which procure many hundreds of millions of doses on behalf of their constituents, must comply with WHO Pre-Qualification (PQ) requirements. The Pan American Health Organization (PAHO), and other procurement organizations may accept the WHO PQ or approvals from certain NRAs such as FDA and EMA. The PQ process, intended to ensure the quality of vaccines, is highly structured and systematic to ensure the manufacturing company’s policies and practices not only produce a product that meets international standards of quality, but also produces a product that meets the programmatic requirements of the national immunization program and WHO use recommendations. Manufacturers must have systems in place to respond to issues that may occur in routine manufacturing and to new safety or field use issues that may arise once the produce is in wide-spread use. A full PQ assessment process can take 8–12 months to complete in addition to the time required for companies to respond to comments [12], [23]. Also, WHO must issue guidance/policy on the use of such a vaccine as part of a national immunization program. Manufacturers must determine if their vaccine is supported by a WHO recommendation for use. If it is not, then the manufacturer will need to engage with the appropriate authorities at WHO to ascertain what data are needed to obtain such a guidance/policy statement, in addition to the data required to show safety, efficacy, and manufacturing quality for PQ. WHO charges fees for vaccine PQ in order to ensure the financial sustainability and quality of the program, and a newly developed WHO PQ fee structure went into effect January 1, 2017, which distinguishes vaccines by complexity and charges higher fees for combination or novel vaccines [19].

Before a vaccine can be considered for PQ, the NRA that is sponsoring the vaccine to WHO PQ must be certified as “functional” for vaccine sponsoring purposes by WHO. WHO takes a risk-based approach to balance appropriate controls with timely access to essential medicines and priority vaccines [23]. While WHO's standards for NRA “functionality” are significant (there are only 2 African states with an NRA that are WHO recognized as “functional for vaccines sponsoring purposes”), WHO actively engages with countries to develop regulatory capacity [23]. As the NRA also regulates clinical trials and certifies GMP, manufacturers depend on clear and timely information and guidance from their NRA. Given the complexity of many vaccines and manufacturing processes, it can take years, if not decades, to build capacity to effectively regulate the vaccine industry at a local level in accordance with international standards.

## 5 Conclusion

Like other technology-driven and highly-regulated industries, vaccine manufacturing is capital-intensive, and long-term product costs are driven primarily by development and production-related economics [24]. Costs of development and maintenance of the production process, construction and operation of manufacturing facilities and compliance with local and international regulations are all incremental to traditional manufacturing costs such as raw material, facilities, maintenance and labor. Achieving large scale production and long product lifecycles help manufacturers produce at low cost and recover their investments in vaccine research and development. In addition, regulatory requirements, including WHO prequalification, local NRA licensure and licensure in the country of use, combined with QA/QC requirements, are significant drivers of cost and require well-trained staff that can adapt to any regulatory changes.

Equipment and skilled labor that are not available in low-resource countries will need to be imported, and maintained or replaced, for years if not decades. Countries seeking to augment or localize vaccine supply will need to invest heavily in facilities, equipment, skilled labor and ongoing quality management with a long time-horizon – requiring “patient capital” and development of in-house technical skills. Countries or companies must also carefully weigh the systemic risks and inherent difficulties in high quality vaccine manufacture and be prepared that returns may only accrue in the long-term, if at all. Industry consolidation and failure of many manufacturers to achieve sustainable returns in the industry suggest that despite the tremendous value of vaccines to global public health and their role in reducing childhood and adult mortality, vaccine manufacturing remains a challenging and costly endeavor.

## Funding

This work was supported by the Bill & Melinda Gates Foundation, Seattle, WA, USA.

## Role of the funding source

The funder of this study had staff and consultants (co-authors of this manuscript) who had a role in study design, data analysis, data interpretation, or writing of the report. The corresponding author had full access to all data in the study and had final responsibility for all content and for the decision to submit for publication.

## Conflict of interest statement

None.
